# miR-539 acts as a tumor suppressor by targeting epidermal growth factor receptor in breast cancer

**DOI:** 10.1038/s41598-018-20431-z

**Published:** 2018-02-01

**Authors:** Jilong Guo, Guohua Gong, Bin Zhang

**Affiliations:** 1 Medicinal Chemistry and Pharmacology Institute, Inner Mongolia University for Nationalities, Tongliao, Inner Mongolia 028000 China; 2 Inner Mongolia Key Laboratory of Mongolian Medicine Pharmacology for Cardio-Cerebral Vascular System, Tongliao, Inner Mongolia 028000 China; 3 Affiliated Hospital of Inner Mongolia University for Nationalities, Institute of Mongolia and Western Medicinaltreatment, Tongliao, Inner Mongolia 028007 China

## Abstract

Breast cancer is the most frequently diagnosed malignancy and the leading cause of cancer-associated death in women worldwide. microRNAs (miRNAs) play critical roles in the cellular processes of breast cancer. However, the crucial roles and underlying mechanisms of miR-539 in breast cancer remain unclear. By RT-qPCR, we found that expression of miR-539 was markedly down-regulated in breast cancer tissues and cell lines compared with that in paired adjacent normal tissues and normal cell lines. The low level of miR-539 expression was positively associated with lymph node metastasis. Furthermore, forced expression of miR-539 inhibited proliferation and migration of breast cancer MDA-MB-231 and MCF7 cells *in vitro* and suppressed tumor growth *in vivo*. Moreover, bioinformatics analysis and luciferase reporter assays indicated that epidermal growth factor receptor (EGFR) was a direct target of miR-539. Over-expression of miR-539 decreased the EGFR mRNA and protein levels in MDA-MB-231 and MCF7 cells. In addition, ectopic over-expression of EGFR partly reversed miR-539-inhibited proliferation as well as migration of MDA-MB-231 and MCF7 cells. Taken together, our results demonstrate that miR-539 functions as a tumor suppressor in breast cancer by downregulating EGFR, supporting the targeting of the novel miR-539/EGFR axis as a potentially effective therapeutic approach for breast cancer.

## Introduction

Breast cancer is the most frequently diagnosed malignancy and leading cause of cancer-related death in women worldwide^1^. Breast cancer tumourigenesis can be described as a multi-step process in which normal cells undergo malignant transformation to a fully developed tumor through accumulation of genetic and epigenetic changes^2,3^. Although alterations in many tumor-suppressor and oncogenic genes have been implicated in breast cancer, the molecular mechanisms that maintain the malignant progression of tumor cells remain poorly understood. Hence, it is essential to elucidate the underlying molecular mechanisms of breast cancer and develop novel strategies for early diagnosis and treatment of patients with breast cancer.

MicroRNAs (miRNAs) are endogenous, small, non-coding molecules of 15∼22 nucleotides; they can bind to the 3′-untranslated regions (3′-UTRs) of target genes to suppress their expression^4–6^. Studies have demonstrated that miRNAs have broad effects on numerous biological processes, including differentiation, metastasis, apoptosis, metabolism and maturation^7,8^. Recently, aberrant expression of miRNAs has been shown to regulate the progression of breast cancer^9^. For example, miR-106b targets FUT6 (Fucosyltransferase 6) to promote cell migration, invasion, and proliferation in human breast cancer^10^. miR-145 suppresses breast cancer cell migration by targeting FSCN-1 (Fascin actin-bundling protein 1) and inhibiting epithelial-mesenchymal transition^11^. miR-183-5p promotes cell proliferation and inhibits apoptosis in human breast cancer by targeting PDCD4 (programmed cell death 4)^12^. miR-206 inhibits the stemness and metastasis of breast cancer by targeting the MKL1/IL11 pathway^13^.

The gene encoding miR-539 is located on human chromosome 4q32.31, and miR-539 has been reported to be down-regulated in many human cancers, including prostate cancer^14^, nasopharyngeal carcinoma^15^ and thyroid cancer^16^. miR-539 has been reported to play a tumor suppression role in many human malignancies^14–17^. However, the biological roles and potential molecular mechanisms of miR-539 in breast cancer have not been elucidated.

In this study, we showed that miR-539 was significantly down-regulated in breast cancer tissues and cell lines compared with paired adjacent normal tissues and normal cell lines and was associated with lymph node metastasis. Over-expression of miR-539 significantly decreased the growth and migration of breast cancer cells *in vitro* and inhibited tumor growth *in vivo*. Notably, we identified that epidermal growth factor receptor (EGFR) was a target of miR-539. Ectopic over-expression of miR-539 suppressed breast cancer cell proliferation and migration via reducing EGFR expression.

## Results

### miR-539 was significantly down-regulated in breast cancer tissues and cell lines

**Figure 1:**
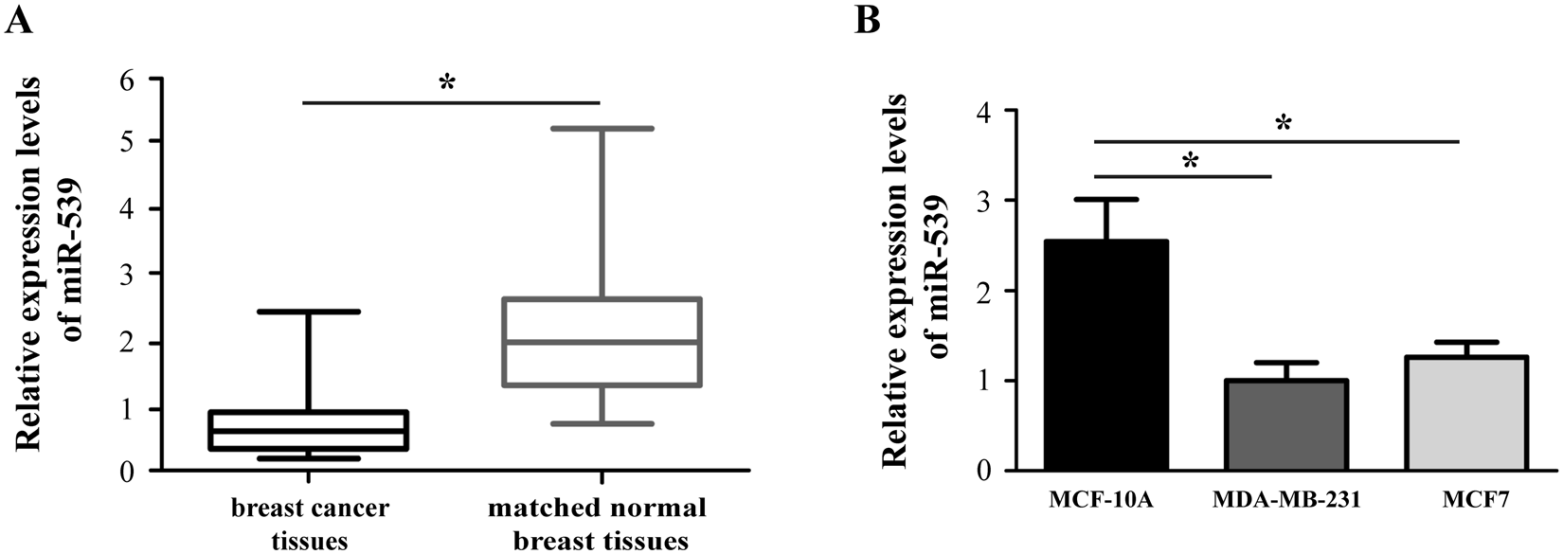
miR-539 was significantly down-regulated in breast cancer tissues and cell lines. (**A**) Relative expression levels of miR-539 in 38 pairs of breast cancer tissues and matched normal breast tissues were analysed by reverse transcription-quantitative polymerase chain reaction (RT-qPCR). (**B**) Relative expression of miR-539 in two breast cancer cell lines (MDA-MB-231 and MCF7) and a human normal mammary epithelial cell line (MCF-10A). miRNA: microRNA. *P < 0.05.

We performed RT-qPCR to examine the miR-539 expression levels in both breast cancer samples and cell lines. Paired breast cancer tissues and normal breast tissues were obtained from 38 patients diagnosed with breast cancer. The results showed that miR-539 expression was significantly down-regulated in the breast cancer tissues compared with that in the matching normal breast tissues (Fig. 1A, P < 0.05). Based on the miR-539 expression levels measured by RT-qPCR, the 38 patients were divided into low and high miR-539 expression groups using the median expression level as the cut-off point (0.51; range: from 0.09 to 2.54). The associations between the miR-539 expression levels and clinical characteristics were evaluated by the chi-square test. The data showed that low miR-539 expression was positively associated with lymph node metastasis (Table 1, P < 0.05) but no significant associations were observed with other parameters, including the age, primary tumor size, histological subtype, AJCC stage, histological grade, distant metastasis, and estrogen receptor.

**Table 1:** Association between the miR-539 expression levels and clinicopathological characteristics of patients with breast cancer.

Variables	Cases	miR-539 expression		P value
		Low (n, %)	High (n, %)	
All patients	38	25 (65.8)	13 (34.2)	
Age (years)				0.307
$<$45	16	12 (31.6)	4 (10.5)	
$\ge 45$	22	13 (34.2)	9 (23.7)	
Primary tumor size (cm)				0.732
$\mathrm{ < 2}$	19	13 (34.2)	6 (15.8)	
$\ge 2$	19	12 (31.6)	7 (18.4)	
Histological subtype				0.324
Invasive ductal carcinoma	32	20 (52.6)	12 (31.6)	
others	6	5 (13.2)	1 (2.6)	
AJCC stage				0.653
I-II	28	19 (50.0)	9 (23.7)	
III-IV	10	6 (15.8)	4 (10.5)	
Histological grade				0.207
G1-G2	20	15 (39.5)	5 (13.2)	
G3	18	10 (26.3)	8 (21.1)	
Lymph node metastasis				0.009
Negative	21	10 (23.6)	11 (28.9)	
Positive	17	15 (39.5)	2 (5.3)	
Distant metastasis				0.192
No	33	23 (60.5)	10 (26.3)	
Yes	5	2 (5.3)	3 (7.9)	
Estrogen receptor				0.510
Negative	12	7 (18.4)	5 (13.2)	
Positive	26	18 (47.4)	8 (21.1)	

Abbreviations: miR, microRNA; AJCC, American Joint Committee on Cancer.

In addition, the expression level of miR-539 was compared between an immortalized nontumorigenic human mammary epithelial cell line (MCF-10A) and 2 well-defined breast cancer cell lines (MDA-MB-231 and MCF7). Analysis of the RT-qPCR results revealed that as for the expression pattern in breast cancer tissues, miR-539 was markedly down-regulated in MDA-MB-231 and MCF7 cells (Fig. 1B, P < 0.05).

### Over-expression of miR-539 suppresses the proliferation of breast cancer cells *in vitro*

**Figure 2:**
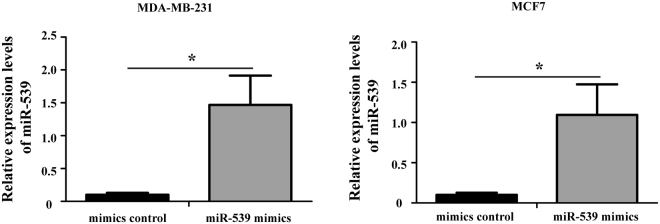
Changes in the expression of miR-539 after transfection with miR-539 mimics or the mimic control. The relative expression levels of miR-539 were evaluated using RT-qPCR. The miR-539 mimics significantly up-regulated the expression levels of miR-539 in MDA-MB-231 and MCF7 cells *P < 0.05.

To evaluate the potential roles of miR-539 in breast cancer cells, we transfected miR-539 mimics or the mimic control into MDA-MB-231 and MCF7 cell lines to produce breast cancer cells with miR-539 over-expression. The data from RT-qPCR confirmed that the MDA-MB-231 and MCF7 cells transfected with miR-539 mimics had significantly higher expression levels of miR-539 than those transduced with the mimic control (Fig. 2, P < 0.05).

**Figure 3:**
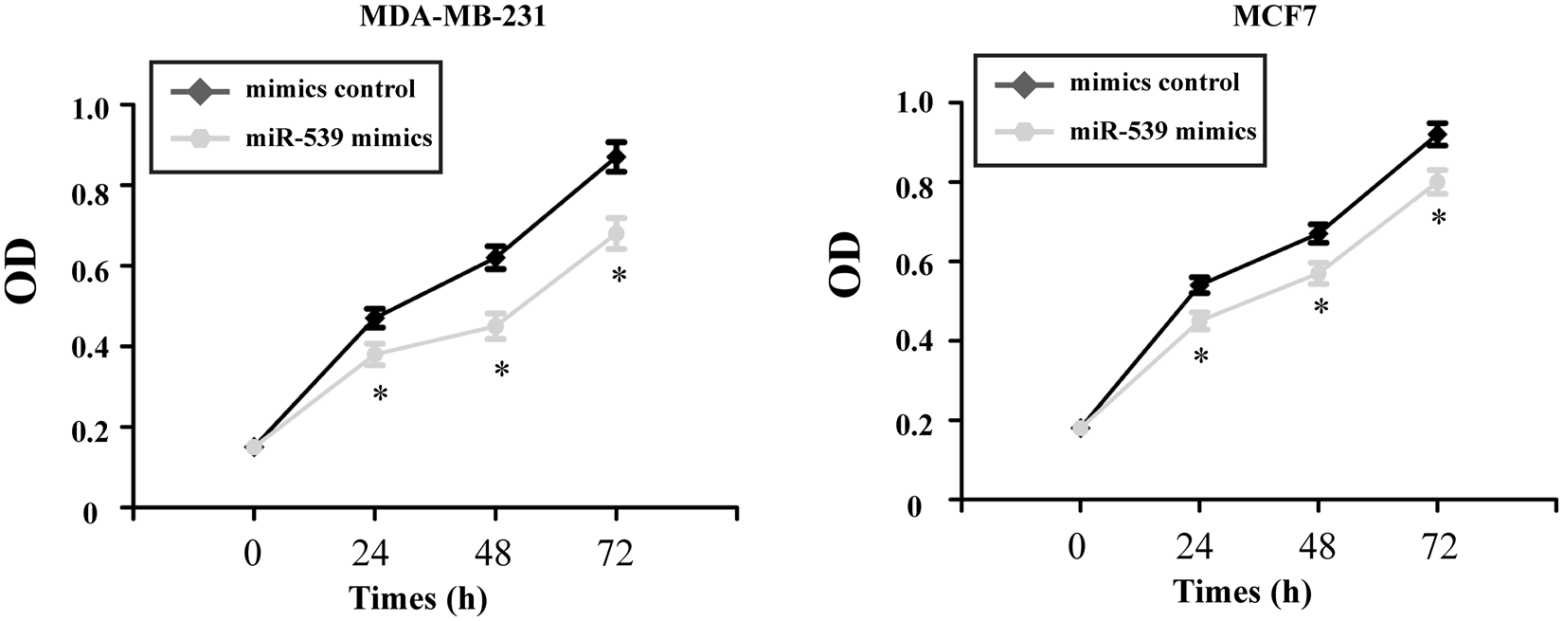
Over-expression of miR-539 suppresses the proliferation of breast cancer cells. Cell proliferation was determined by the MTT assay after transfection with miR-539 mimics or the mimic control. MTT: 3-(4,5-dimethyl-2-thiazolyl)-2,5-diphenyl-2-H-tetrazolium bromide. *P < 0.05.

The MTT assay was used to quantitate the proliferation of the transfected breast cancer cells. The data showed that over-expression of miR-539 significantly suppressed the proliferation of MDA-MB-231 and MCF7 cells compared to the mimic control transfected cells (Fig. 3, P < 0.05).

### miR-539 up-regulation inhibited the migration of breast cancer cells ***in vitro***

**Figure 4:**
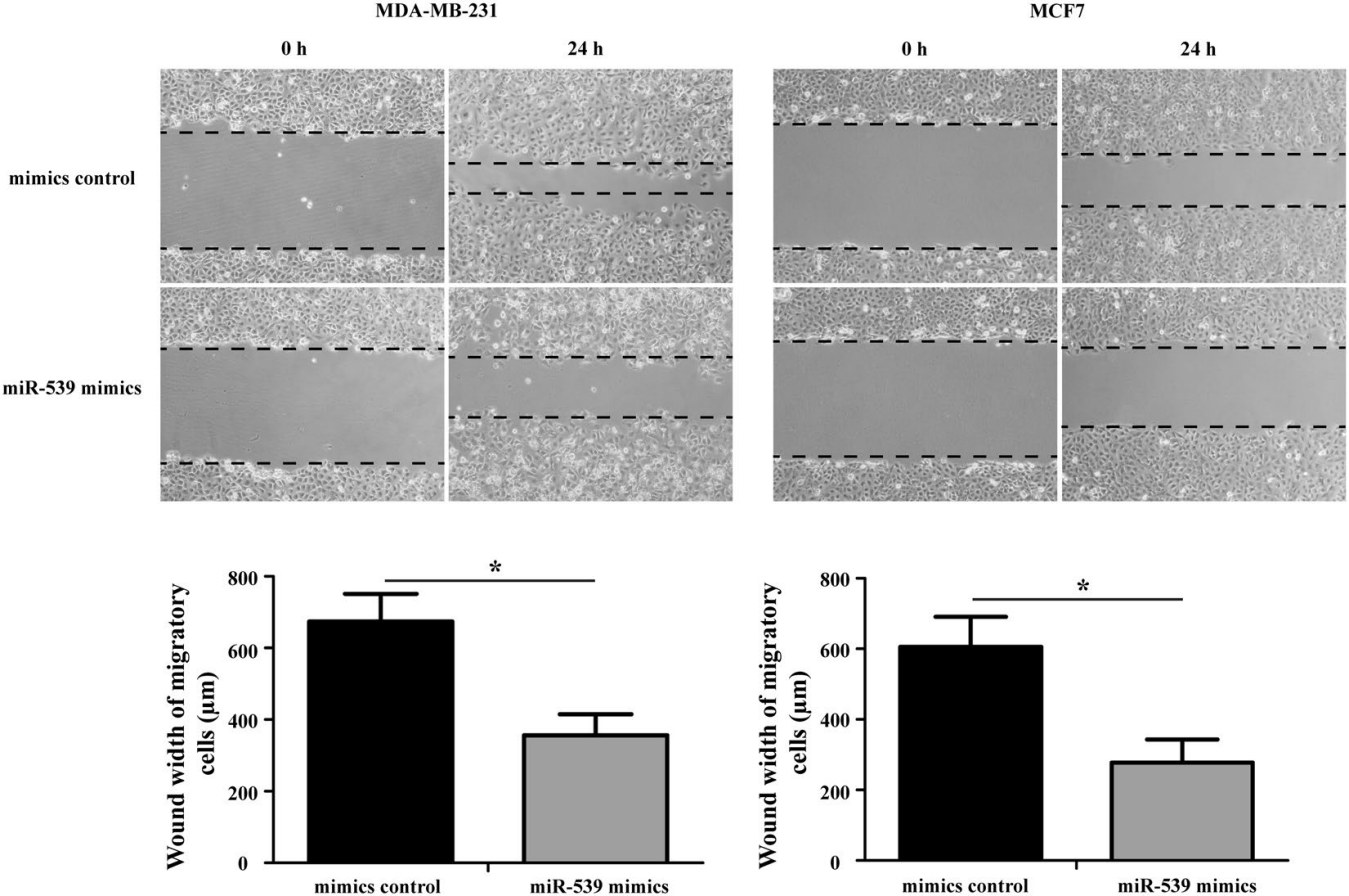
miR-539 up-regulation inhibited the migration of breast cancer cells. Cell migration was determined at 0 h and 24 h in MDA-MB-231 and MCF7 cells via the wound healing assay after treatment with miR-539 mimics or the mimic control (magnification, 200×). *P < 0.05.

A wound healing assay was performed to evaluate the potential role of miR-539 in the migration of breast cancer cells. As shown in Fig. 4, a significant decrease in migration was observed in the miR-539 mimic-transfected MDA-MB-231 and MCF7 cells compared with that in the mimic control-transfected cells (P < 0.05).

### Forced expression of miR-539 suppre**s**ses tumor growth in nude mice

**Figure 5:**
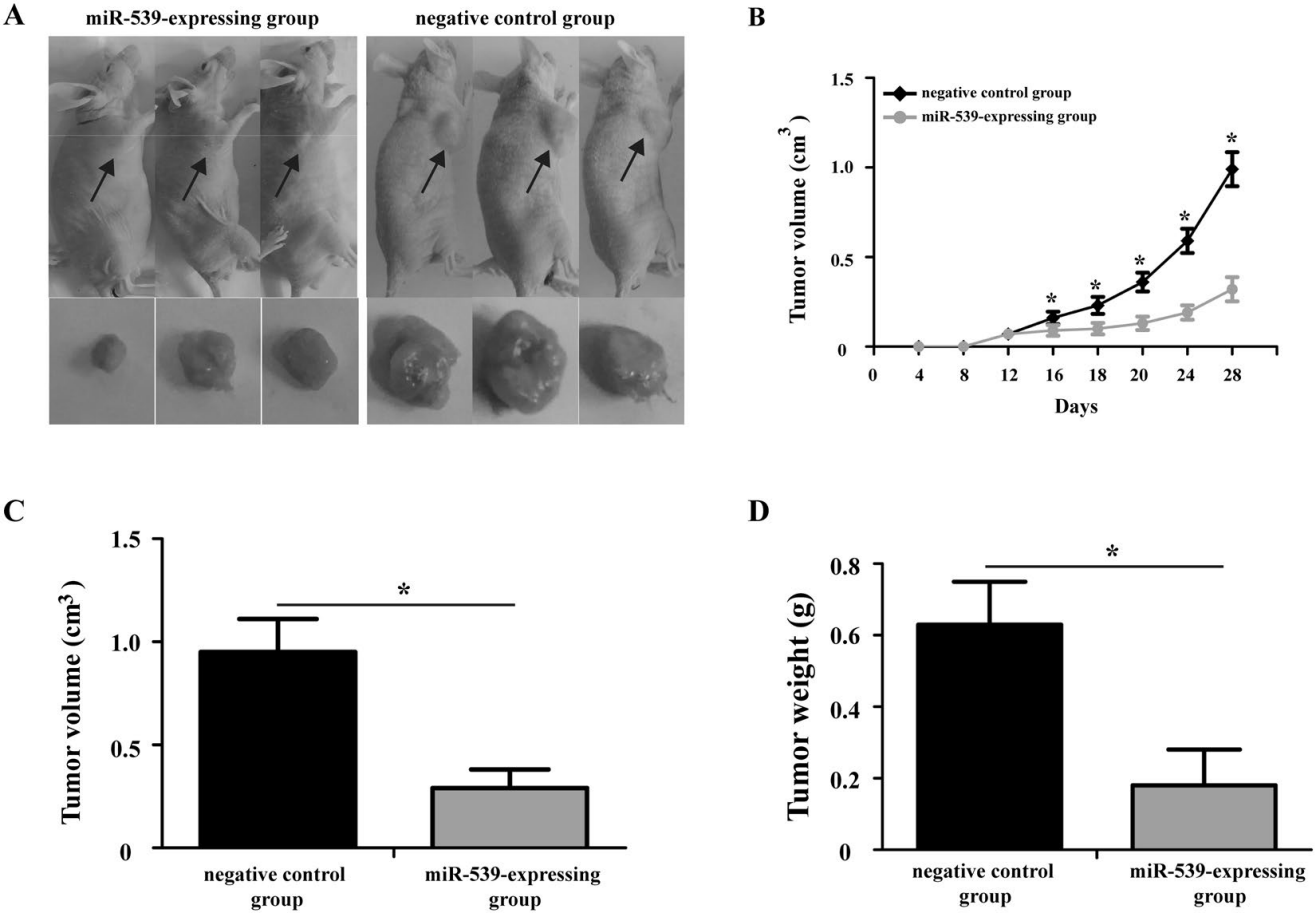
Enforced expression of miR-539 suppresses tumor growth in nude mice. (**A**) Representative images of the mice carrying tumors are shown after 28 days of inoculation with MDA-MB-231 cells expressing miR-539 or negative control. (**B**) Measurement of tumor volumes at the indicated time points. Measurement of the final volume (**C**) and weight (**D**) of xenograft model at day 28. *P < 0.05.

To examine the effect of miR-539 on tumor growth *in vivo*, MDA-MB-231 cells overexpressing miR-539 or negative control were subcutaneously injected into the right flanks of nude mice and tumor growth was closely monitored. Representative images of the mice carrying tumors after 28 days are shown in Fig. 5A. Tumor growth in the miR-539-expressing group was markedly slower than that of the negative control group (Fig. 5B, P < 0.05). Moreover, the final tumor volume (Fig. 5C, P < 0.05) and weight (Fig. 5D, P < 0.05) of the miR-539-expressing group were significantly lower than that of the negative control group.

### EGFR was a direct target gene of miR-539

**Figure 6:**
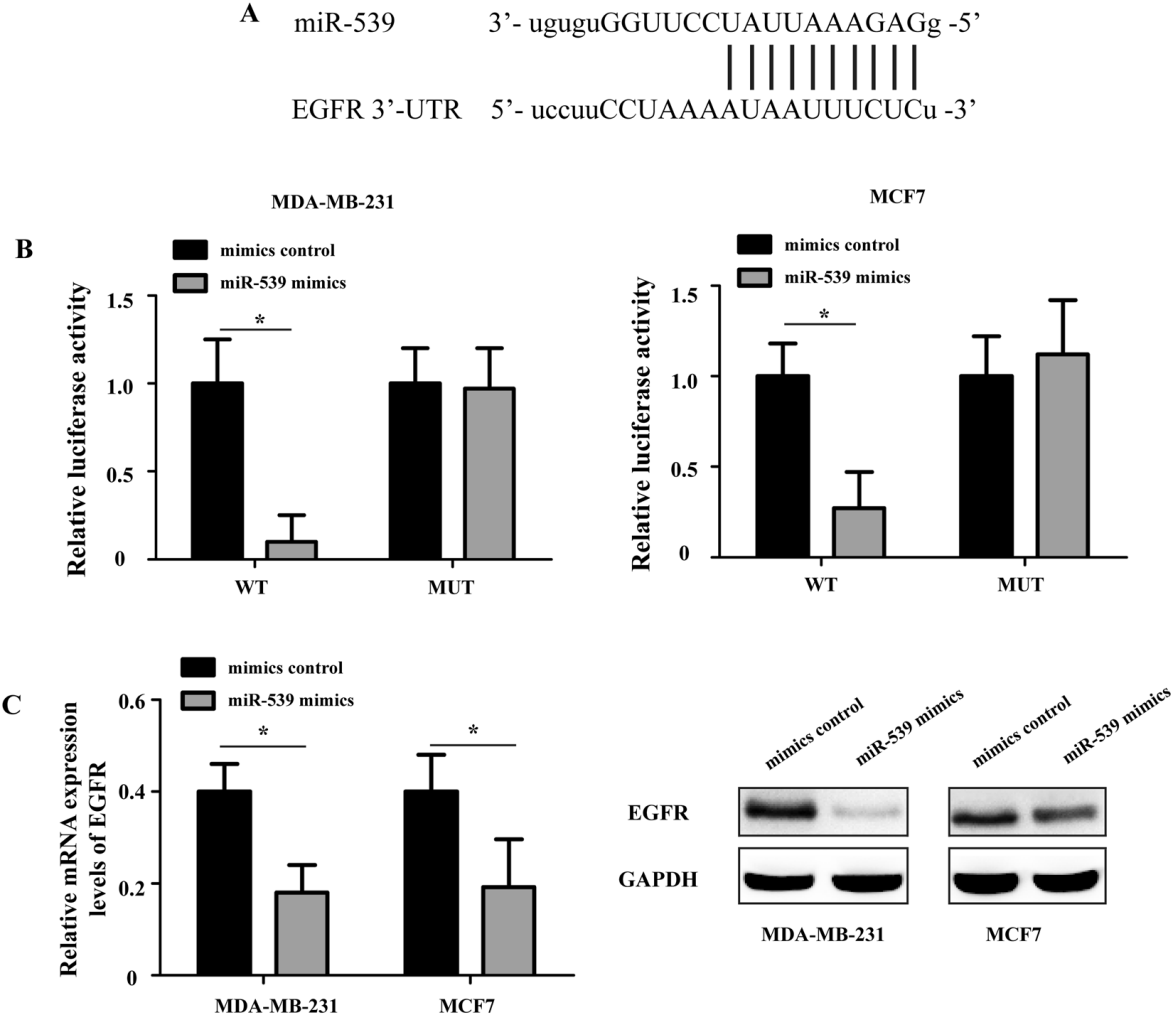
EGFR is a direct target gene of miR-539. (**A**) Bioinformatics analysis of the interaction between miR-539 and its binding site within the 3′-UTR of EGFR. (**B**) Relative luciferase activity in different groups after co-transfection with psiCHECK-2-EGFR (WT) or (MUT) and miR-539 mimics or mimic control. (**C**) The introduction of miR-539 reduced the endogenous EGFR mRNA and protein levels in MDA-MB-231 and MCF7 cells. UTR: untranslated region; EGFR: epidermal growth factor receptor; WT: wild type; and MUT: mutant. *P < 0.05.

To elucidate the potential molecular mechanism underlying the miR-539-mediated regulation of growth and migration, bioinformatics analysis based on computer-aided algorithms (PicTar, TargetScan and miRDB) was performed to predict the target genes of miR-539. The most promising candidate gene was EGFR, which was confirmed by all algorithms. As shown in Fig. 6A, there was a putative 7-mer-binding site for miR-539 in the 3′-UTR of the EGFR mRNA.

To identify whether miR-539 could directly regulate EGFR, a dual-luciferase reporter assay was performed. Luciferase reporter plasmids containing the wild-type 3′-UTR of EGFR (WT) or mutant 3′-UTR of EGFR (MUT) were constructed to verify the binding between miR-539 and EGFR. As shown in Fig. 6B, the MDA-MB-231 and MCF7 cells transfected with miR-539 mimics displayed a significant decrease in relative luciferase activity with the WT luciferase reporter plasmid (P < 0.05); however, the relative luciferase activity of the MUT luciferase reporter plasmid remained unaffected. In addition, transfection of MDA-MB-231 and MCF7 cells with miR-539 mimics significantly decreased the mRNA and protein expression levels of EGFR compared to the corresponding levels in the mimic control-transfected cells (Fig. 6C, P < 0.05). These observations indicated that miR-539 directly targeted EGFR by binding to the complementary region in the 3′-UTR of its mRNA.

### Ectopic over-expression of EGFR partly reversed the miR-539-inhibited proliferation and migration of breast cancer cells *in vitro*

**Figure 7:**
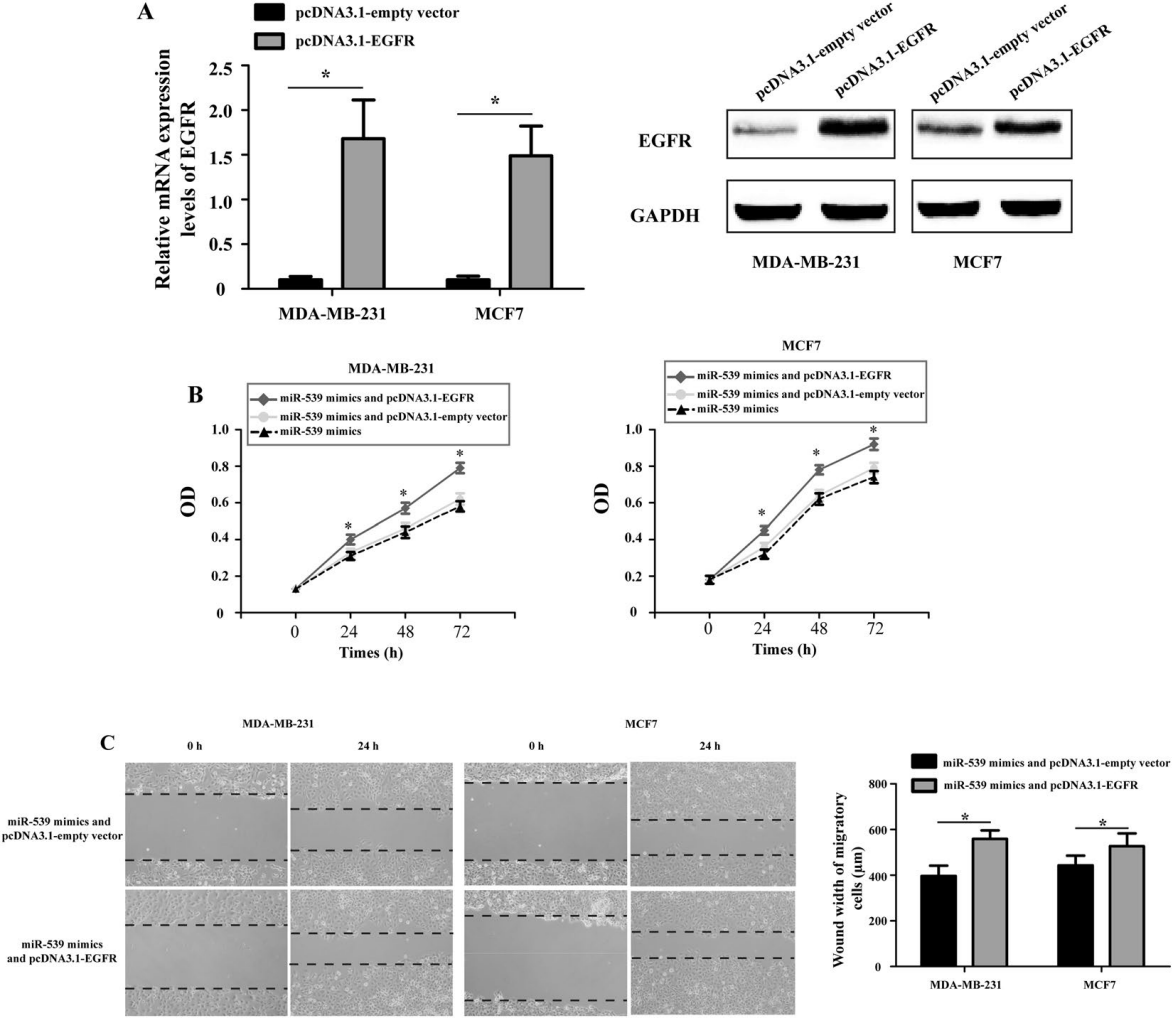
Ectopic over-expression of EGFR partially reversed miR-539-inhibited proliferation and migration of breast cancer cells. (**A**) The relative expression levels of EGFR in MDA-MB-231 and MCF7 cells were analyzed by RT-qPCR and Western blot. (**B**) The proliferation of MDA-MB-231 and MCF7 cells was measured by the MTT assay after miR-539 mimics or miR-539 mimics and pcDNA3.1-EGFR plasmid or miR-539 mimics and pcDNA3.1-empty vector infection. (**C**) A wound healing assay was measured and compared between MDA-MB-231 and MCF7 cells transfected with miR-539 mimics and pcDNA3.1-EGFR plasmid and cells treated with miR-539 mimics and pcDNA3.1-empty vector (Magnification x 200). *P < 0.05.

To confirm whether miR-539 exerted its tumor suppression role through EGFR, EGFR was ectopically expressed using an over-expression plasmid (pcDNA3.1-EGFR). As shown in Fig. 7A, transfection with the pcDNA3.1-EGFR plasmid significantly increased the expression level of EGFR in MDA-MB-231 and MCF7 cells compared with that in the pcDNA3.1-empty vector-transfected cells. MTT and wound healing assays were used to assess the proliferation and migration in MDA-MB-231 and MCF7 cells following transfection with miR-539 mimics and pcDNA3.1-EGFR plasmid or pcDNA3.1-empty vector. The growth of MDA-MB-231 and MCF7 cells significantly increased after co-transfection with miR-539 mimics and pcDNA3.1-EGFR plasmid compared to the cells co-transfected with miR-539 mimics and pcDNA3.1-empty vector (Fig. 7B, P < 0.05). There was no difference between the miR-539 mimic-transfected cells and cells co-transfected with miR-539 mimics and pcDNA3.1-empty vector. The migratory capacity of MDA-MB-231 and MCF7 cells was also markedly increased after co-transfection with miR-539 mimics and pcDNA3.1-EGFR plasmid compared to cells co-transfected with miR-539 mimics and pcDNA3.1-empty vector (Fig. 7C, P < 0.05). These results indicate that forced expression of miR-539 suppressed breast cancer cell proliferation and migration through reducing EGFR expression.

## Discussion

Recent studies have demonstrated that miRNAs act as crucial regulators of gene expression at the transcriptional and/or post-transcriptional level and control a wide range of physiological and pathological processes^6^. Deregulated miRNAs function as either tumor suppressors or oncogenes to regulate the development and progression of most human cancers^18–20^. Increasing evidence has shown that some miRNAs can be used as prognostic biomarkers as well as potential therapeutic targets in breast cancer. For instance, Zhong *et al*. showed that miR-4653-3p and its target gene FRS2 are prognostic biomarkers for hormone receptor-positive breast cancer patients receiving tamoxifen as an adjuvant endocrine therapy^21^. Xu *et al*. found that miR-148a functions to suppress metastasis and serves as a prognostic indicator in triple-negative breast cancer^22^. Dong *et al*. reported that decreased expression of miR-124 is an independent predictor of unfavorable prognosis for patients with breast cancer^23^. Eissa *et al*. identified miR-10b and minichromosome maintenance complex component 5 gene as prognostic biomarkers in breast cancer^24^.

miR-539 has been reported to be down-regulated and to act as a tumor suppressor in many human cancers^14–16^. In the present study, RT-qPCR analysis of clinical samples from 38 breast cancer patients and two breast cancer cell lines demonstrated that miR-539 was significantly down-regulated in breast cancer tissues and cell lines. The low level of miR-539 expression was positively associated with lymph node metastasis. The presence of decreased miR-539 expression in breast cancer was consistent with results from previous reports. To the best of our knowledge, this study is the first to determine the miR-539 expression pattern in breast cancer. These data suggest that miR-539 may play an important role in regulating breast cancer progression.

Subsequently, the biological functions of miR-539 were examined in two breast cancer cell lines (MDA-MB-231 and MCF7). The data from the MTT and wound healing assays and tumor xenograft model demonstrated that up-regulation of miR-539 significantly suppressed breast cancer cell proliferation and invasion *in vitro* as well as and suppressed tumor growth *in vivo*, confirming the findings from previous studies and suggesting a tumor suppressive role for this miRNA. These results provide substantial evidence that miR-539 is involved in the malignant progression of breast cancer.

Previous studies have found that sperm-associated antigen 5 (SPAG5)^14^, cyclin-dependent kinase 4 (CDK4)^25^, and caspase recruitment domain family member 11 (CARMA1)^16^ are targets of miR-539 in some human cancers. Here, to explore the molecular mechanism by which miR-539 suppresses the progression of breast cancer, we identified EGFR as a direct target gene of miR-539. EGFR is a member of the ERBB cell-surface receptor tyrosine kinase family^26^. EGFR is of immediate medical and biological importance because of its well-established roles in developmental biology, tissue homeostasis and cancer^27^. It has been reported that dysregulation of EGFR enhances metastasis in many solid cancers, such as lung cancer, breast cancer and osteosarcoma^28^. Over-expression of EGFR has been reported in 15–20% of all breast carcinomas and in 50–70% of triple-negative breast cancers (TNBC)^29,30^. It is known that breast cancer patients with high EGFR expression have tumors that are more aggressive, larger and more likely to metastasize to lymph nodes^26^. Additionally, patients with EGFR-positive tumors have worse overall, disease free and post-relapse survival after hormonal and/or chemotherapy^26^.

Bioinformatics analysis based on computer-aided algorithms has shown that there is a putative 7-mer-binding site for miR-539 on the 3′-UTR of EGFR mRNA. Here, by using the dual-luciferase reporter assay, we confirmed that EGFR is a direct target of miR-539. Over-expression of miR-539 significantly suppressed the expression of EGFR at both the mRNA and protein levels in MDA-MB-231 and MCF7 cells. In addition, ectopic over-expression of EGFR partially reversed the miR-539-inhibited proliferation and migration of breast cancer cells. Taken together, these results indicate that EGFR is a direct, functional target of miR-539 in breast cancer.

In conclusion, our results demonstrated, for the first time, that miR-539 expression was down-regulated in breast cancer tissues and cell lines. Over-expression of miR-539 suppressed cell proliferation and migration *in vitro* and suppressed tumor growth *in vivo*. Moreover, we identified EGFR as a direct target gene of miR-539. Over-expression of miR-539 suppressed breast cancer cell proliferation and migration by reducing EGFR expression. These findings indicate that miR-539 functions as a tumor suppressor in breast cancer at least partially by regulating EGFR, suggesting that miR-539 may be a promising target for treating breast cancer in the future.

## Materials and Methods

### Breast cancer tissue specimens

This study was conducted with informed consent from all the patients before we performed our experiments. All studies involving human subjects were also approved by the Ethics Committee of Inner Mongolia University for Nationalities, and they were performed in accordance with the regulations of Declaration of Helsinki. Thirty-eight paired breast cancer tissue specimens and matching normal breast tissue samples (located 2.0 cm outside the primary tumor site) were obtained from Inner Mongolia University for Nationalities between May 2010 and May 2015. None of the patients had received preoperative treatment, such as radiotherapy or chemotherapy. All samples were immediately flash-frozen in liquid nitrogen and stored at −80 °C until further use. The average age of patients was 46.5 $\pm$ 5.7 years. Detailed clinicopathological characteristics of patients with breast cancer are shown in Table 1. All tissues were histopathologically examined by hematoxylin-eosin (HE) staining.

### Cell lines and cell culture

Human breast cancer cell lines (MDA-MB-231 and MCF7) and an immortalized non-tumor human mammary epithelial cell line (MCF-10A) were obtained from the Chinese Academy of Sciences (Shanghai, China). The MDA-MB-231 and MCF7 cells were cultured in DMEM (Invitrogen, Carlsbad, CA, USA) supplemented with 10% FBS (Invitrogen, Carlsbad, CA, USA) containing streptomycin (100 mg/ml) and penicillin (100 U/ml) and maintained in an incubator at 37 °C and 5% CO_2_. The MCF-10A cells were cultured in DME/F12 medium (Invitrogen, Carlsbad, CA, USA) containing 5% FBS, 20 ng/ml EGF, 10 $\mu$g/ml insulin, 100 ng/ml cholera toxin, 0.5 $\mu$g/ml hydrocortisone, 100 U/ml penicillin and 100 $\mu$g/ml streptomycin.

### Plasmid constructs, RNA oligonucleotides and cell transfection

The ORF (open reading frame) of EGFR was PCR-amplified from the human cDNA library and cloned into the pcDNA3.1-vector to obtain the EGFR over-expression plasmid (pcDNA3.1-EGFR). The sequences of pcDNA3.1-EGFR were confirmed by Sanger sequencing. miR-539 mimics and the mimic control were purchased from Shanghai GenePharma, Co. Ltd., (Shanghai, China).

MDA-MB-231 and MCF7 cells were cultured in 6-well plates until they reached 75∼85% confluence. Transfections were performed using Lipofectamine 2000 (Invitrogen, Carlsbad, CA, USA) according to the manufacturer’s protocols. For each well, 50 pmol of miR-539 mimics and the mimic control or 4 *μ*g of pcDNA3.1-EGFR and pcDNA3.1-empty vectors were added to 500 *μ*l of DMEM with 5 *μ*l Lipofectamine 2000. The mixture was added to each well and incubated for 48 h at 37 °C and 5% CO_2_. Total RNA and/or protein was extracted for reverse transcription-quantitative polymerase chain reaction (RT-qPCR) and/or Western blot analysis.

### RNA isolation and reverse transcription-quantitative polymerase chain reaction (RT-qPCR)

The total RNA of breast cancer cells and tissues was isolated using TRIzol reagent (Invitrogen, Carlsbad, CA, USA) according to the manufacturer’s instructions. A Reverse Transcription Kit (Invitrogen, Carlsbad, CA, USA) was used to convert total RNA into cDNA. qPCR was performed on an ABI PRISM 7500 Sequence Detection System (Applied Biosystems, CA, USA) using a miScript SYBR Green PCR Kit (Qiagen, Germany) and SYBR Premix Ex Taq Kit (TaKaRa, Japan) to determine the expression levels of miR-539 and EGFR. The qPCR conditions consisted of 30 min of DNA polymerase activation at 95 °C, which was followed by 40 cycles at 95 °C for 10 sec and 60 °C for 30 sec. Glyceraldehyde-3-phosphate dehydrogenase (GAPDH) and U6 small nuclear RNA were used as two internal references for normalization. The sequences of primers used for amplification are as follows: miR-539: 5′-GGAGAAATTATCCTTGGTGTGT-3′ (forward), universal primer (reverse); U6: 5′-CTCGCTTCGGCAGCACATA-3′ (forward), 5′-CGAATTTGCGTGTCATCCT-3′ (reverse); EGFR: 5′-AAGGCACGAGTAACAAGC-3′ (forward), 5′-AGGGCAATGAGGACATAA-3′ (reverse); and GAPDH: 5′-TCAAGAAGGTGGTGAAGCA-3′ (forward), 5′-AGGTGGAGGAGTGGGTGT-3′ (reverse). The relative mRNA and miRNA expression levels were calculated using the delta–delta Ct method^31^.

### Western blot analysis

Cells were collected and extracted by RIPA buffer (Invitrogen, Carlsbad, CA, USA) on ice. A BCA Protein Assay Kit (Pierce, IL, USA) was used to determine the concentration of each sample. Approximately 50 $\mu$g of total protein was separated by 12% SDS-PAGE (Beyotime, Shanghai, China) and then blotted onto PVDF (Millipore, MA, USA) membranes. The membranes were then blocked with 5% non-fat milk for 2 h at 37 °C and incubated with anti-EGFR antibody (1:500; CST Technologies, Inc., Chicago, IL, USA) and anti-GAPDH antibody (1:1000; CST Technologies, Inc., Chicago, IL, USA) overnight at 4 °C. After the membranes were washed with TBST for 5 min, they were incubated with horseradish peroxidase-conjugated (HRP) goat anti-mouse or anti-rabbit IgG (1:2000; CST Technologies, Inc., Chicago, IL, USA) for 1 h at 37 °C. Positive bands were detected using an ECL Western blot detection kit (GE Healthcare, Pittsburgh, PA, USA) according to the manufacturer’s protocols.

### Bioinformatics analysis

Online target gene analysis software miRDB (http://www.mirdb.org), TargetScan (http://www.targetscan.org) and PicTar (http://www.pictar.org) were used to detect complementary sequences for binding between miR-539 and EGFR.

### Dual-luciferase reporter assay

The 3′-UTR of human EGFR, including the complementary binding site of miR-539, was cloned downstream of the firefly cassette of the psiCHECK-2 dual-luciferase reporter plasmid (Promega, WI, USA) to construct the psiCHECK-2-EGFR wild-type (WT) luciferase reporter plasmid. The complementary binding site was then mutated using a site-directed gene mutagenesis kit (TaKaRa, Japan). The mutated EGFR 3′-UTR was also inserted into the psiCHECK-2 plasmid to generate the psiCHECK-2-EGFR mutant (MUT) luciferase reporter plasmid. MDA-MB-231 and MCF7 cells were co-transfected with psiCHECK-2-EGFR (WT) or (MUT) and miR-539 mimics or the mimic control using Lipofectamine 2000 according to the manufacturer’s protocols. Forty-eight hours after transfection, a dual-luciferase reporter assay system (Promega, WI, USA) was used to detected the firefly and *Renilla* luciferase activities. The firefly luciferase activities were normalized to the *Renilla* luciferase activities.

### Cell proliferation assay

Cell proliferation was examined using the MTT (3-(4,5-dimethyl-2-thiazolyl)-2,5-diphenyl-2-H-tetrazolium bromide) assay. Briefly, approximately 6000 MDA-MB-231 or MCF7 cells/well were seeded into 96-well plates. After 24 h, the cells were transfected with miR-539 mimics or the mimic control for 48 h and then treated with 25 μl/well MTT (5 mg/ml; Sigma, CA, USA). After incubation for 4 h at 37 °C, the supernatant was discarded and the formazan products were dissolved with dimethyl sulfoxide (DMSO; Sigma, CA, USA). Finally, the optical density (OD) at 480 nm was detected using a microplate reader (Synergy HT Multi-Mode Microplate Reader; Biotek, Winooski, VT, USA).

### Cell migration assay

Cell migration was assessed with the wound healing assay. Briefly, MDA-MB-231 or MCF7 cells (4 × 10^5^/well) were seeded in 6-well plates. At 24 h after transient transfection, an artificial wound was created onto the monolayer using a sterile 100-*μ*l tip. After scratching, the cells were washed three times with PBS and incubated at 37 °C with 5% CO_2_. Images of cell migration were captured after 0 and 12 h under a light microscope (ECLIPSE TS100; Nikon Corporation, Tokyo, Japan).

### Tumor xenograft model

A total of 30, four-week-old, male nude mice were obtained from Model Animal Research Center Of Nanjing University (Nanjing, China). All mice were maintained under specific pathogen-free (SPF) conditions at ∼22 °C under a 12 h light/dark cycle and had free access to food and water. All animal experiments were performed with the approval of the Ethic Committee of Inner Mongolia University for Nationalities.

Lentiviral vectors overexpressing miR-539 were constructed according to a previously described method^14^. To establish a xenograft tumor model, MDA-MB-231 cells stably expressing miR-539 or negative control were subcutaneously injected into the right flanks of nude mice that were 4∼5 weeks of age. Tumor volumes were measured every four days using the formula volume (mm^3^) = (width^2^ × length)/2. Mice were killed 28 days after injection under anesthesia; then, the final tumor volumes were measured and the tumors were weighed.

### Statistical analysis

SPSS software version 17.0 (SPSS, Chicago, IL, USA) was used for statistical analysis. The data were represented as the means ± standard deviation (SD). The Chi-square test was used to analyze the relationship between miR-539 and clinicopathologic parameters. One-way ANOVA (analysis of variance) or Student’s t-test was used to analyze differences between groups. A value of P < 0.05 was considered statistically significant. All experiments were performed in triplicate for biological replicates.

## Electronic supplementary material

Supplementary figure 1
