# Supplementary material for: miR-539 acts as a tumor suppressor by targeting epidermal growth factor receptor in breast cancer
Source: Sci Rep. 2018 Feb 1;8:2073. doi: 10.1038/s41598-018-20431-z (PMC5794864; doi:10.1038/s41598-018-20431-z)

Supplementary Figure Legend

Tumour-suppressive miR-539 inhibits tumour cell growth and migration by directly targeting EGFR in breast cancer

Authors: Jilong Guo, Guohua Gong, Bin Zhang

Supplementary figure 1. Analysis of EGFR expression in MDA-MB-231 and MCF7 cells by Western blot. (A) Introduction of miR-539 reduced the endogenous EGFR protein expression in MDA-MB-231 and MCF7 cells. (B) The levels of EGFR was markedly increased in both MDA-MB-231 and MCF7 cells after transfected with pcDNA3.1-EGFR plasmid.

A

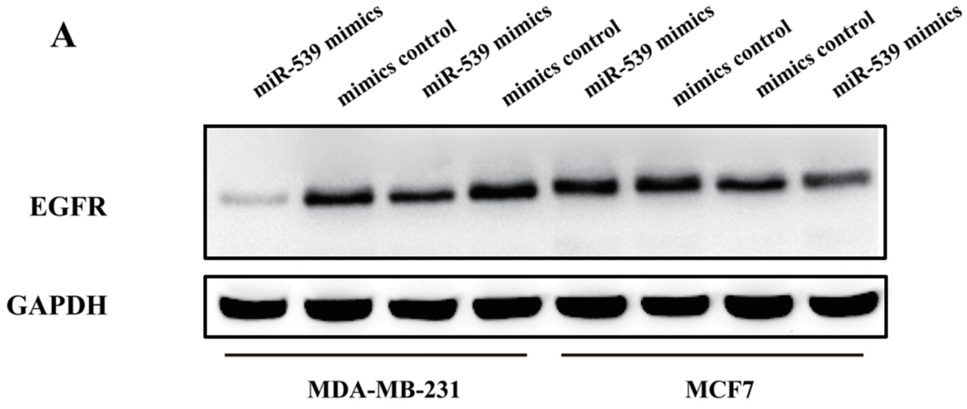

B

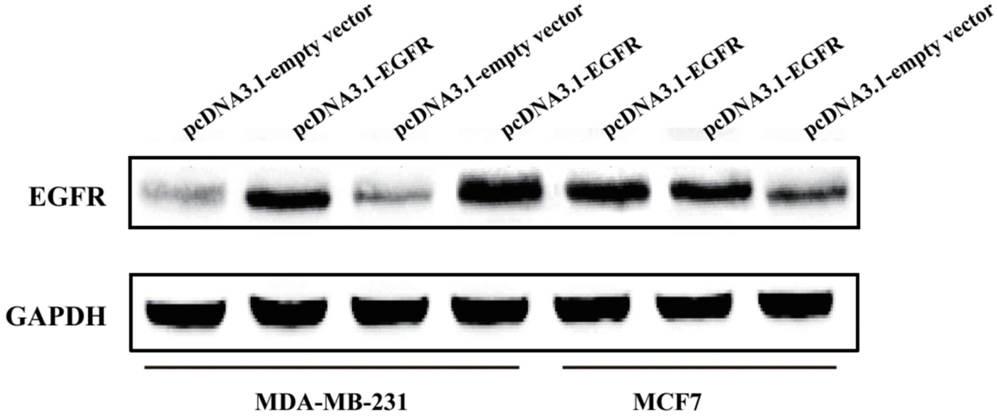

Supplement: Supplementary file 1 — Supplementary figure 1 [file 41598_2018_20431_MOESM1_ESM.pdf]
